# Novel Oral Treatment of Anastomotic Stenosis After Salvage Extended Sleeve Lobectomy

**DOI:** 10.1016/j.atssr.2025.09.029

**Published:** 2025-10-29

**Authors:** Kana Kinjo, Shinsuke Uchida, Aritoshi Hattori, Mariko Fukui, Takeshi Matsunaga, Kazuya Takamochi, Kenji Suzuki

**Affiliations:** Department of General Thoracic Surgery, Juntendo University School of Medicine, Tokyo, Japan

## Abstract

Treatment of anastomotic stenosis after bronchoplasty varies, depending on the degree of stenosis. This report describes the case of a 66-year-old man who experienced anastomotic stenosis after salvage-extended double-sleeve lobectomy after chemoradiation and immunotherapy for recurrent lung cancer. The patient was treated with oral tranilast and clarithromycin, which resulted in significant improvement in the stenosis within 4 weeks. This case highlights the potential efficacy of antiinflammatory medications in managing anastomotic stenosis and preventing granulation tissue formation.

Management of anastomotic stenosis after bronchoplasty includes various options; however, no standardized treatment protocol exists. Although bronchoscopic and laser interventions are common treatment techniques, studies on conservative approaches, such as oral medications, remain limited. Physical interventions for stenosis, including bronchoscopic procedures, can be burdensome for patients in terms of their frequency and duration.^1^ Here we report on the effectiveness of a noninvasive, conservative treatment method with oral medications in the management of a patient with anastomotic stenosis.

A 66-year-old man had received a diagnosis of squamous cell carcinoma in the upper lobe of the left lung (cT3 N2 M0, stage IIIB) by bronchoscopic biopsy at another institution. His medical history included diabetes mellitus (hemoglobin A1c, 6.9%) and rheumatoid arthritis, which was managed without steroid therapy. He had a 30-pack-year history of smoking. The patient underwent definitive chemoradiotherapy comprising carboplatin and paclitaxel combined with radiotherapy (60 Gy/30 fractions), followed by durvalumab, and achieved a partial response.

Three months later, computed tomography (CT) revealed 2 recurrent tumors in the left upper lobe of the lungs. Second-line chemotherapy with docetaxel and ramucirumab failed to control tumor growth, thus prompting a referral to our hospital (Juntendo University School of Medicine, Tokyo, Japan) for salvage surgery. Preoperative CT revealed 20- and 16-mm pure-solid nodules in the left S3 and S1+2 lung segments, respectively (Figures 1A, 1B). Radiation pneumonitis was evident on the mediastinal side from the left upper lobe to the S6 lung segment, with narrowing of the upper lobe bronchi and B6 resulting from, previous radiotherapy. Posterolateral thoracotomy revealed scarring in the surrounding lung tissues of the left upper lobe and S6 segment after radiotherapy. Additionally, the deformed bronchi in the left upper lobe and S6 segment necessitated left upper lobectomy and S6 segmentectomy, with bronchopulmonary artery plasty to preserve the left basal segment. Bronchoplasty was performed between the left main and left basal bronchi by using a running suture technique with 4-0 polypropylene (Prolene, Ethicon) suture and a double-ended needle. Simultaneously, pulmonary artery plasty was performed between the left main and basal pulmonary arteries by using the running suture technique with 6-0 Prolene. Pathologic examination confirmed the squamous cell carcinoma (ypT3 N0 M0 stage IIB, major pathological response). The proximal and peripheral bronchi were negative for malignancy, and the percentage of residual tumor was 12.5%. No endobronchial components were observed in the resected tissue. The patient had lower pulmonary function postoperatively, but no additional treatment was administered. Bronchoscopy performed on postoperative day (POD) 6 revealed favorable anastomotic findings (Figure 1C). Recovery was uneventful, and the patient was discharged on POD 9. Slight ischemic changes of anastomosis were revealed on POD 20 (Figure 1D).

**Figure 1:**
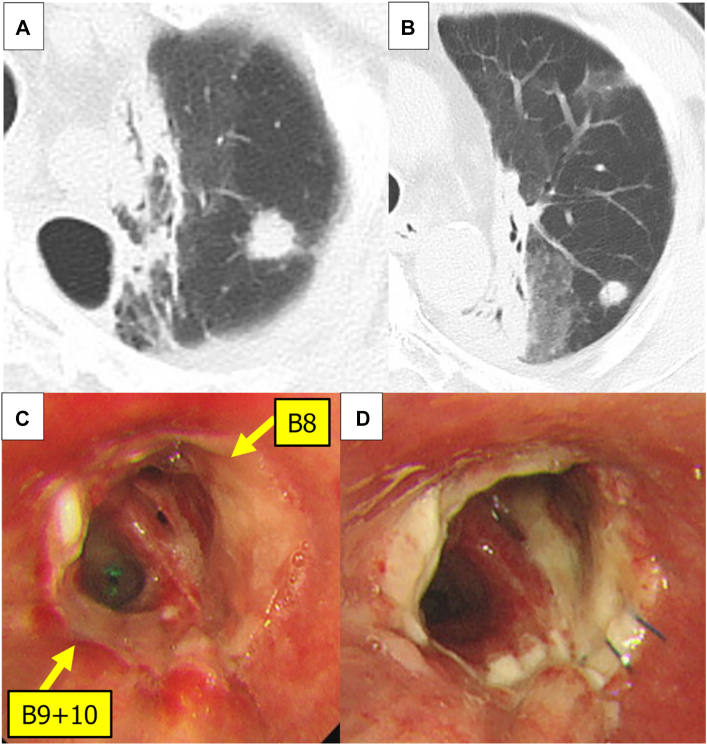
(A) A pure-solid nodule in the left S3 segment. (B) A pure-solid nodule in the left S1+2 segment. (C) Anastomotic findings on postoperative day 6. B8 indicates the bronchus of segment 8, and B9+10 indicates the bronchi of segments 9 and 10. (D) Anastomotic findings on postoperative day 20.

On POD 27, bronchoscopy identified circumferential ischemic changes (Figure 2A) that progressed to anastomotic stenosis caused by granulation tissue formation by POD 48 (Figure 2B). The patient was asymptomatic; however, treatment was initiated because of a likely progressive stenosis. The patient was prescribed oral tranilast and clarithromycin, thereby targeting granulation tissue with their expected antiinflammatory effects. As anticipated, the stenosis improved significantly by POD 76 (Figure 2C), and further improvement was observed by POD 125 (Figure 2D). No restenosis or side effects were observed after 11 weeks of oral administration of tranilast and clarithromycin.

**Figure 2:**
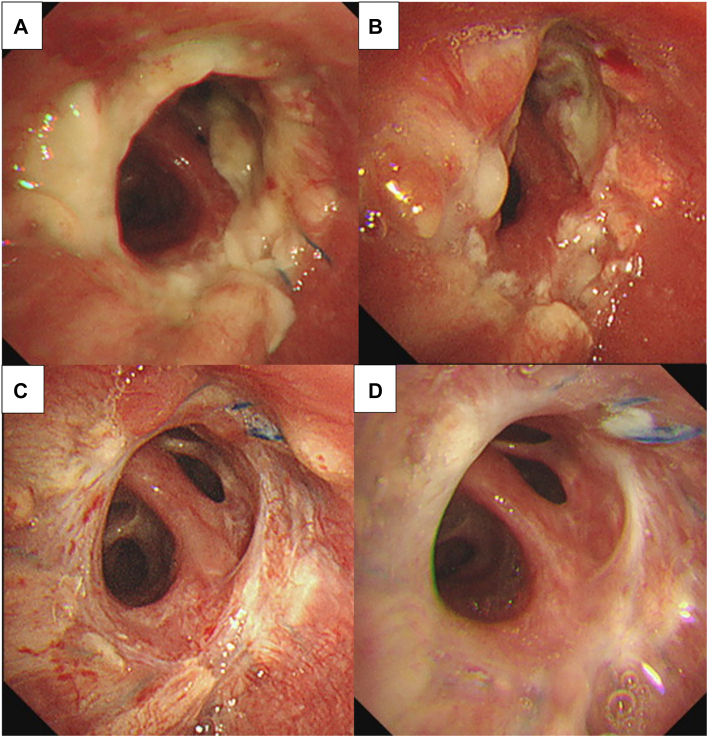
(A) Anastomotic findings with ischemic changes on postoperative day 27. (B) Anastomotic findings with stenosis on postoperative day 48. (C) Anastomotic findings on postoperative day 76. (D) Anastomotic findings without stenosis on postoperative day 125.

## Comment

The incidence of anastomotic stenosis after sleeve lobectomy is between 1% and 4%.^2^ Anastomotic stenosis treatment strategies often vary among institutions. Although mechanical interventions, such as bougies and stenting, have been documented, internal therapies are less fully explored.

Adequate bronchial circulation with well-oxygenated blood is critical for anastomotic wound healing. New blood vessels form between the proximal and peripheral bronchial sides after 2 weeks postoperatively, stabilizing the anastomosis by completing blood circulation around it.^3^ However, prolonged periods of insufficient blood flow can induce inflammatory cellular infiltration, leading to ischemic changes. These changes trigger fibroblast activity, resulting in granulation tissue formation and eventual scarring around the anastomosis, thereby causing stenosis.^4^

The standard treatments for anastomotic stenosis include bronchoscopic balloon catheter dilatation,^5^ stent placement, laser therapy, steroidal therapy, and reoperation. However, no established treatment guidelines exist for managing this condition, and immediate complete resolution is uncommon, even after multiple interventions in several cases.

In this case, oral administration of tranilast and clarithromycin significantly improved the anastomotic stenosis. Tranilast—an allergy-modulating agent commonly used for hypertrophic and keloid scars in plastic surgery—suppresses fibrocyte proliferation, which increases when mast cells and eosinophils secrete excessive allergic mediators.^6^ Clarithromycin, beyond its antimicrobial function, mitigates chronic neutrophilic airway inflammation by decreasing cytotoxic agents, thus making it effective against diffused panbronchiolitis.^7^ These agents, in combination, reduce the inflammation caused by eosinophils and neutrophils, alleviate ischemia, and improve blood flow, thereby facilitating the resolution of granulation tissue and subsequently, anastomotic stenosis. Tranilast causes cystitis and impaired liver function in rare cases. However, these medications are prescription drugs without severe adverse effects.^6^ No disadvantages of the oral treatment were observed in this case.

**Figure 3:**
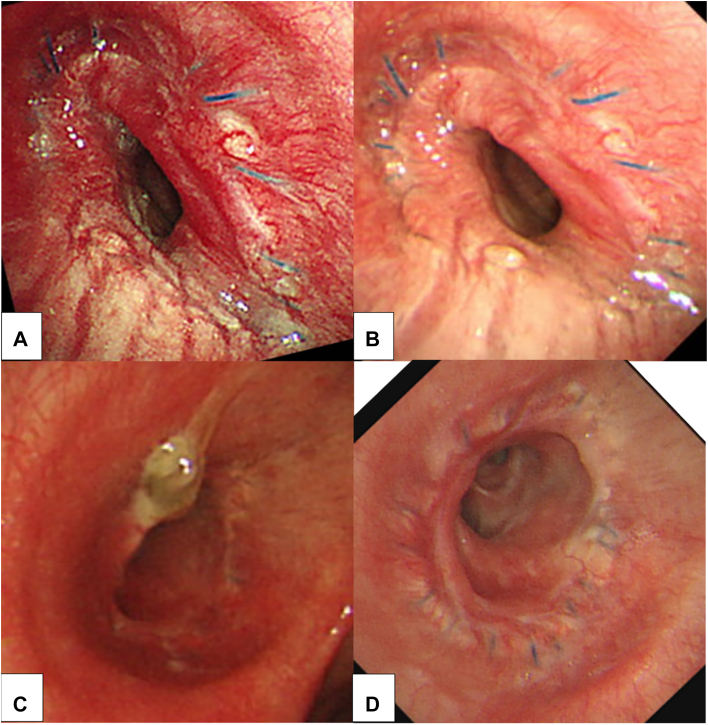
In the first more recent patient, (A) anastomotic findings before taking tranilast and clarithromycin. and (B) anastomotic findings after oral medications. In the second more recent patient, (C) anastomosis findings before taking tranilast and clarithromycin and (D) anastomotic findings after oral medication use.

At our institution, 2 additional patients recently received tranilast and clarithromycin for anastomotic stenosis after right upper sleeve lobectomy. In the first patient, the medications were initiated 5 months postoperatively (Figure 3A), resulting in improved inflammatory findings within 4 weeks (Figure 3B). The medications were discontinued after 12 weeks. In the second patient, the medications were initiated 2 months postoperatively (Figure 3C), and a significant improvement was observed after 4 weeks (Figure 3D). A comparison of these 3 cases suggests that different responses to treatment depend on the timing of medication initiation; thus, it is inferred that early administration of these 2 drugs before granulation is fully developed may be more effective.

In conclusion, oral administration of tranilast and clarithromycin proved effective in our patient; therefore, these drugs should be considered as a prestage treatment before interventions such as balloon catheter dilatation or stent placement. Noninvasive oral treatment initiated at the early stage of asymptomatic granulation formation at the anastomosis may prevent the need for invasive interventions. Antiinflammatory drugs offer noninvasive treatment for the management of anastomotic stenosis.
